# National Trends in the Association of Race and Ethnicity With Predialysis Nephrology Care in the United States From 2005 to 2015

**DOI:** 10.1001/jamanetworkopen.2020.15003

**Published:** 2020-08-27

**Authors:** Tanjala S. Purnell, Sunjae Bae, Xun Luo, Morgan Johnson, Deidra C. Crews, Lisa A. Cooper, Macey L. Henderson, Raquel C. Greer, Sylvia E. Rosas, L. Ebony Boulware, Dorry L. Segev

**Affiliations:** 1 Division of Transplantation, Department of Surgery, Johns Hopkins School of Medicine, Baltimore, Maryland; 2 Department of Epidemiology, Johns Hopkins Bloomberg School of Public Health, Baltimore, Maryland; 3 Johns Hopkins Center for Health Equity, The Johns Hopkins University, Baltimore, Maryland; 4 Division of Nephrology, Department of Medicine, Johns Hopkins School of Medicine, Baltimore, Maryland; 5 Division of General Internal Medicine, Johns Hopkins School of Medicine, Baltimore, Maryland; 6 Kidney and Hypertension Unit, Joslin Diabetes Center and Harvard Medical School, Boston, Massachusetts; 7 Division of General Internal Medicine, Duke University School of Medicine, Durham, North Carolina

## Abstract

**Importance:**

Predialysis nephrology care is associated with better survival among patients with end-stage kidney disease.

**Objective:**

To examine national trends in racial/ethnic disparities in receipt of predialysis nephrology care at least 1 year before dialysis initiation in the United States from 2005 to 2015.

**Design, Setting, and Participants:**

This national registry study assessed US registry data of 1 000 390 adults in the US Renal Data System who initiated maintenance dialysis treatment from January 1, 2005, to December 31, 2015, in multiple cross-sectional analyses. Multivariable logistic regression models were used to examine national trends in racial/ethnic disparities in receipt of predialysis nephrology care with adjustments for potential confounders. Data were analyzed April 17, 2020.

**Exposure:**

Race/ethnicity of the patients.

**Main Outcomes and Measures:**

Receipt of at least 12 months of predialysis nephrology care as determined by clinician-based documentation on the End Stage Renal Disease Medical Evidence Report Form CMS 2728.

**Results:**

Among 1 000 390 adults (57.2% male; 54.6% White, 27.8% Black, 14.0% Hispanic, and 3.6% Asian; mean [SD] age, 62.4 [15.6] years) who initiated maintenance dialysis in the United States from 2005 to 2015, 310 743 (31.1%) received at least 12 months of predialysis nephrology care. In 2005 to 2007, compared with White adults, the adjusted odds ratio for receipt of at least 12 months of predialysis nephrology care was 0.82 (95% CI, 0.80-0.84) among Black adults, 0.67 (95% CI, 0.65-0.69) among Hispanic adults, and 0.84 (95% CI, 0.80-0.89) among Asian adults; in 2014 to 2015, the adjusted odds ratio was 0.76 (95% CI, 0.74-0.78) among Black adults, 0.61 (95% CI, 0.60-0.63) among Hispanic adults, and 0.90 (95% CI: 0.86-0.95) among Asian adults.

**Conclusions and Relevance:**

In this cross-sectional study of more than 1 million US adults with end-stage kidney disease, racial and ethnic disparities in predialysis nephrology care did not substantially improve from 2005 to 2015. Study findings suggest that national strategies to address racial/ethnic disparities in predialysis nephrology care are needed.

## Key Points

**Question:**

Have racial and ethnic disparities in the receipt of at least 12 months of predialysis nephrology care narrowed during the last decade in the United States?

**Findings:**

In this cross-sectional study of national registry data for 1 000 390 US adults with end-stage kidney disease, receipt of at least 12 months of predialysis nephrology care increased overall from 2005 to 2015; however, disparities did not improve. In 2005 to 2007, compared with White adults, the odds of receiving predialysis nephrology care was lower by 18% among Black adults, 33% among Hispanic adults, and 16% among Asian adults; similar differences were observed in 2014 to 2015 (24% among Black adults, 39% among Hispanic adults, and 10% among Asian adults).

**Meaning:**

This national study found that racial and ethnic disparities in receipt of at least 12 months of predialysis nephrology care did not improve from 2005 to 2015, suggesting that national strategies to address these disparities are needed.

## Teaser

This national registry study examines national trends in racial/ethnic disparities in the receipt of predialysis nephrology care at least 1 year before dialysis initiation among adults with end-stage kidney disease in the United States from 2005 to 2015.

## Introduction

Receipt of nephrology care before the initiation of treatment for end-stage kidney disease (ESKD) is associated with improved patient survival and other key outcomes, including reduced hospitalizations and complications, increased quality of life, better preparation for dialysis, and greater likelihood of receiving a kidney transplant.^1,2,3^ Primary care clinicians are generally the first to detect chronic kidney disease (CKD) and play key roles in deciding when to refer patients to nephrology care.^4^ Current guidelines recommend referral to a nephrologist for patients with an estimated glomerular filtration rate of less than 30 mL/min/1.73 m^2^, severely increased albuminuria, rapid decline of the estimated glomerular filtration rate, hematuria, and/or uncontrolled complications of CKD, such as hypertension requiring 4 or more antihypertensive agents, anemia, or electrolyte abnormalities.^5^ However, late referral to nephrology care remains common, with recent reports^6^ suggesting that approximately one-third of patients with CKD receive nephrology care at least 12 months before the start of ESKD therapy.

Clinicians are also advised to refer their patients with CKD to a nephrologist if they are part of a demographic group known to experience more rapid kidney disease progression, such as young adults and racial/ethnic minorities.^4^ However, prior studies^7,8,9,10,11^ suggest that racial/ethnic disparities exist in timely receipt of nephrology care. Prakash et al^7^ reported that Black patients with CKD are less likely to receive a timely referral to a nephrologist than White patients, and patients living in areas with larger populations of Black residents are less likely than those living in other areas. Non-White patients are also more likely to be referred to a nephrologist during later stages of CKD than their White counterparts.^7^ Postulated reasons for these disparities include differences in insurance, socioeconomic status, educational attainment, provider biases, and geographic barriers.^7,8,9,10,11^

The Healthy People 2020 (HP2020) initiative, coordinated by the US Department of Health and Human Services, provides a vision and strategy for improving the health of all US residents by setting priorities, identifying baseline data and 10-year targets for specific objectives, monitoring outcomes, and evaluating progress.^12^ The HP2020 CKD-specific objectives were designed to monitor and assess efforts to reduce the long-term burden of kidney disease, increase lifespan, improve quality of life, and eliminate related health care disparities.^12^ A key HP2020 CKD objective is to “increase the proportion of chronic kidney disease patients receiving care from a nephrologist at least 12 months before the start of renal replacement therapy.”^12^ In addition, 1 of the 4 overarching goals of HP2020 is to eliminate health care disparities.^12^ As such, the primary goal of the present study was to examine national trends and assess whether racial/ethnic disparities in receipt of at least 12 months of predialysis nephrology care narrowed during the last decade in the United States.

## Methods

### Data Sources

This cross-sectional study followed the Strengthening the Reporting of Observational Studies in Epidemiology (STROBE) reporting guideline.^13^ The study used data from the US Renal Data System (USRDS), a national data system that collects, analyzes, and distributes information about ESKD in the United States. Staff of the USRDS collaborate with staff from the Centers for Medicare & Medicaid Services (CMS), the United Network for Organ Sharing, and the End Stage Renal Disease (ESRD) networks to share data sets and actively work to improve the accuracy of patient information. The study was reviewed by the institutional review board at the Johns Hopkins University School of Medicine and determined to qualify for an exemption under the Protection of Human Participants (45 CFR §46.101[b] [2006]) because study participants cannot be identified directly or through linked identifiers; this exemption precluded the need for informed consent owing to the use of deidentified data.

### Main Outcomes and Measures

The study population included adults (aged ≥18 years) who initiated maintenance dialysis in the United States from January 1, 2005, to December 31, 2015, as captured in the USRDS. The primary study outcome, receipt of at least 12 months of predialysis nephrology care, was defined as response of yes and a marked checkbox for greater than 12 months by clinician documentation on the ESRD Medical Evidence Report Form CMS 2728 in response to the question, “Prior to ESRD therapy, was patient under care of a nephrologist? If yes, answer: <6 months, 6-12 months, or >12 months.” We limited the study population to adults initiating dialysis on or after January 1, 2005, because the USRDS did not ascertain receipt of predialysis nephrology care before 2005. Patients were excluded if they were younger than 18 years, if their ESRD Medical Evidence Report Form CMS 2728 was missing, or if data for race or ethnicity were missing. Patients designated as 1 of the fixed USRDS racial/ethnic categories of non-Hispanic White, non-Hispanic Black, non-Hispanic Asian, or Hispanic (any race) were included in the study. This study was not sufficiently powered to examine outcomes among patients identified as American Indian/Alaska Native, Pacific Islander, Middle Eastern/Arabian, Indian (subcontinent), or other/unknown owing to the small population sizes in some study years. The date of the analysis being reported was April 17, 2020.

### Statistical Analysis

#### Descriptive Data Analysis

Clinical and demographic characteristics were stratified by patient race/ethnicity and year of dialysis initiation. Wilcoxon rank sum (for continuous variables) and χ^2^ (for categorical variables) tests were performed to compare distributions and assess statistical significance.

#### Regression Models

Associations between race/ethnicity and receipt of at least 12 months of predialysis nephrology care were analyzed using both univariable (estimating crude odds ratios [ORs] with 95% CIs) and multivariable (estimating adjusted ORs [aORs] with 95% CIs) logistic regression models. Data were categorized into multiyear increments (2005-2007, 2008-2010, 2011-2013, and 2014-2015) based on the date of patient initiation of maintenance dialysis to allow for an adequate sample size in each analytical cell. Multivariable regression models were adjusted for biologically plausible confounders (age, sex, body mass index [calculated as weight in kilograms divided by height in meters squared], and ESKD etiology). Statistical interaction terms were used in regression models to formally test the statistical significance of temporal changes in racial/ethnic disparities in receipt of predialysis nephrology care.

#### Mediation Analyses

We performed subsequent multivariable logistic regression models to examine the extent to which racial/ethnic differences in the following potential mediators attenuated disparities in predialysis nephrology care: (1) comorbid medical conditions (ie, cancer, atherosclerotic heart disease, congestive heart failure, diabetes, hypertension, chronic obstructive pulmonary disease, or peripheral vascular disease), (2) health insurance type, and (3) type of dialysis modality and type of vascular access. To estimate the influence of these potential mediators on primary study outcomes, these factors were incrementally incorporated into the main multivariable logistic regression models (adjusting for age, sex, body mass index, and ESKD etiology).

#### Sensitivity Analyses

To test the robustness of our study findings, we repeated primary multivariable logistic regression models to assess racial/ethnic differences in receipt of predialysis nephrology care at any time before ESKD therapy. Finally, we repeated primary analyses using modified Poisson regression models (estimating adjusted risk ratios) to further test the robustness of our primary study findings.

#### Model Testing and Statistical Significance

Missing variable levels were modeled separately from known variable levels in regression models. The robustness of estimates was tested by comparing results from an alternate modeling approach to handle missing data (multiple imputation), and inferences remained the same for the study outcomes of interest. Two-tailed *P* < .05 was considered statistically significant. All analyses were conducted using Stata, version 16/MP for Linux (StataCorp LLC).

## Results

### Study Population

A total of 1 218 610 adults aged 18 years or older were documented in the USRDS as having initiated maintenance dialysis in the United States from January 1, 2005, to December 31, 2015. For the study analysis, we excluded 25 654 patients (2.1%) owing to incongruent or missing race/ethnicity, 51 (0.004%) owing to missing sex, 10 948 (0.9%) owing to missing body mass index, 30 (0.002%) owing to missing health insurance type, and 181 537 (14.9%) owing to missing nephrology care variables. These exclusions resulted in 1 000 390 adults for the final analysis (428 542 female [42.8%] and 571 848 male [57.2%]; mean [SD] age, 62.4 [15.6] years) (Figure).

**Figure.**
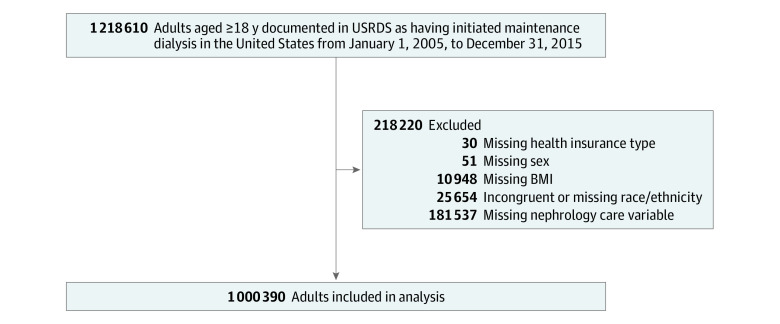
Study Population Flow Diagram BMI indicates body mass index; USRDS, US Renal Data System.

### Patient Characteristics

Among the 1 000 390 eligible adults (546 132 White [54.6%], 278 317 Black [27.8%], 139 854 Hispanic [14.0%], and 36 087 Asian [3.6%]) who initiated maintenance dialysis in the United States from 2005 to 2015, 310 743 (31.1%) received at least 12 months of predialysis nephrology care. Mean age remained relatively consistent across the study period, whereas we found increased body mass index (>34.9, 43 187 [17.3%] in the 2005-2007 cohort vs 36 036 [21.0%] in the 2014-2015 cohort) and increased prevalence of male patients (139 620 [56.0%] in the 2005-2007 cohort vs 99 560 [58.1%] in the 2014-2015 cohort). Black and Hispanic patients were younger and less likely to have private insurance than Asian and White patients. The prevalence of ESKD attributed to hypertension or diabetes was highest among Black and Hispanic patients, whereas the prevalence of ESKD attributed to glomerular diseases or other causes was highest among White and Asian patients (Table 1 and Table 2).

**Table 1.** Characteristics of Adults Who Initiated ESKD Treatment in the US in the 2005-2007 and 2008-2010 Cohorts^a^

Characteristic	2005-2007 Cohort				2008-2010 Cohort			
	White (n = 137 991)	Black (n = 70 772)	Hispanic (n = 32 792)	Asian (n = 7673)	White (n = 158 683)	Black (n = 82 091)	Hispanic (n = 40 978)	Asian (n = 10 168)
Age, mean (SD), y	65.6 (15.2)	57.4 (15.5)	58.4 (15.8)	62.1 (16.0)	65.7 (15.1)	57.9 (15.3)	58.3 (15.7)	62.4 (16.0)
Age, y								
18-44	14 025 (10.2)	14 705 (20.8)	6241 (19.0)	1138 (14.8)	15 298 (9.6)	16 056 (19.6)	7688 (18.8)	1470 (14.5)
45-60	32 055 (23.2)	25 285 (35.7)	10 894 (33.2)	2067 (26.9)	36 786 (23.2)	29 442 (35.9)	13 987 (34.1)	2703 (26.6)
>60	91 911 (66.6)	30 782 (43.5)	15 657 (47.7)	4468 (58.2)	106 599 (67.2)	36 593 (44.6)	19 303 (47.1)	5995 (59.0)
Female	57 304 (41.5)	34 580 (48.9)	14 230 (43.4)	3494 (45.5)	64 620 (40.7)	38 934 (47.4)	17 284 (42.2)	4472 (44.0)
BMI								
<30.0	89 901 (65.1)	43 681 (61.7)	22 497 (68.6)	6621 (86.3)	97 394 (61.4)	47 882 (58.3)	26 824 (65.5)	8594 (84.5)
30.0-34.9	24 217 (17.5)	12 798 (18.1)	5646 (17.2)	680 (8.9)	29 553 (18.6)	15 416 (18.8)	7562 (18.5)	989 (9.7)
>34.9	23 873 (17.3)	14 293 (20.2)	4649 (14.2)	372 (4.8)	31 736 (20.0)	18 793 (22.9)	6592 (16.1)	585 (5.8)
Cause of ESKD								
Diabetes	56 471 (40.9)	30 210 (42.7)	19 700 (60.1)	3641 (47.5)	63 695 (40.1)	35 243 (42.9)	24 812 (60.5)	4904 (48.2)
Hypertension	35 844 (26.0)	24 390 (34.5)	5724 (17.5)	1779 (23.2)	42 450 (26.8)	29 493 (35.9)	7654 (18.7)	2551 (25.1)
Glomerular diseases	10 327 (7.5)	4633 (6.5)	2203 (6.7)	921 (12.0)	10 986 (6.9)	4615 (5.6)	2607 (6.4)	1171 (11.5)
Other causes	35 349 (25.6)	11 539 (16.3)	5165 (15.8)	1332 (17.4)	41 552 (26.2)	12 740 (15.5)	5905 (14.4)	1542 (15.2)
Health insurance								
Private	88 607 (64.2)	28 406 (40.1)	12 621 (38.5)	3794 (49.4)	99 179 (62.5)	33 902 (41.3)	16 063 (39.2)	4953 (48.7)
Medicare	34 225 (24.8)	22 622 (32.0)	9599 (29.3)	2175 (28.3)	40 974 (25.8)	25 310 (30.8)	11 372 (27.8)	2995 (29.5)
Medicaid	9390 (6.8)	11 845 (16.7)	6402 (19.5)	1167 (15.2)	11 614 (7.3)	13 961 (17.0)	8194 (20.0)	1505 (14.8)
Other	5769 (4.2)	7899 (11.2)	4170 (12.7)	537 (7.0)	6916 (4.4)	8918 (10.9)	5349 (13.1)	715 (7.0)
Received nephrology care for >12 mo before dialysis	41 587 (30.1)	17 338 (24.5)	6968 (21.2)	2003 (26.1)	51 921 (32.7)	21 426 (26.1)	9265 (22.6)	2964 (29.2)

Abbreviations: BMI, body mass index (calculated as weight in kilograms divided by height in meters squared); ESKD, end-stage kidney disease.

^a^ Unless otherwise indicated, data are expressed as number (percentage) of patients. Percentages have been rounded and may not total 100.

**Table 2.** Characteristics of Adults Who Initiated ESKD Treatment in the US in the 2011-2013 and 2014-2015 Cohorts^a^

Characteristic	2011-2013 Cohort				2014-2015 Cohort			
	White (n = 155 763)	Black (n = 79 532)	Hispanic (n = 41 532)	Asian (n = 11 195)	White (n = 93 695)	Black (n = 45 922)	Hispanic (n = 24 552)	Asian (n = 7051)
Age, mean (SD), y	65.5 (14.8)	58.3 (15.1)	58.8 (15.4)	62.6 (16.1)	65.3 (14.6)	58.6 (14.9)	59.3 (15.1)	63.1 (15.7)
Age, y								
18-44	14 384 (9.2)	14 670 (18.4)	7237 (17.4)	1619 (14.5)	8723 (9.3)	8352 (18.2)	3985 (16.2)	934 (13.2)
45-60	36 700 (23.6)	27 825 (35.0)	14 215 (34.2)	2921 (26.1)	22 102 (23.6)	15 651 (34.1)	8386 (34.2)	1826 (25.9)
>60	104 679 (67.2)	37 037 (46.6)	20 080 (48.3)	6655 (59.4)	62 870 (67.1)	21 919 (47.7)	12 181 (49.6)	4291 (60.9)
Female	62 575 (40.2)	37 254 (46.8)	17 124 (41.2)	5011 (44.8)	37 290 (39.8)	21 441 (46.7)	9951 (40.5)	2978 (42.2)
BMI								
<30.0	92 242 (59.2)	45 045 (56.6)	26 393 (63.5)	9319 (83.2)	54 122 (57.8)	26 025 (56.7)	15 467 (63.0)	5849 (83.0)
30.0-34.9	30 477 (19.6)	15 772 (19.8)	8114 (19.5)	1223 (10.9)	19 088 (20.4)	9038 (19.7)	4833 (19.7)	762 (10.8)
>34.9	33 044 (21.2)	18 715 (23.5)	7025 (16.9)	653 (5.8)	20 485 (21.9)	10 859 (23.6)	4252 (17.3)	440 (6.2)
Cause of ESKD								
Diabetes	64 784 (41.6)	34 084 (42.9)	25 452 (61.3)	5559 (49.7)	40 656 (43.4)	19 763 (43.0)	15 245 (62.1)	3607 (51.2)
Hypertension	42 464 (27.3)	30 106 (37.9)	8069 (19.4)	2841 (25.4)	25 589 (27.3)	17 853 (38.9)	4963 (20.2)	1783 (25.3)
Glomerular diseases	10 319 (6.6)	4088 (5.1)	2364 (5.7)	1222 (10.9)	6087 (6.5)	2204 (4.8)	1351 (5.5)	721 (10.2)
Other causes	38 196 (24.5)	11 254 (14.2)	5647 (13.6)	1573 (14.1)	21 363 (22.8)	6102 (13.3)	2993 (12.2)	940 (13.3)
Health insurance								
Private	89 677 (57.6)	31 479 (39.6)	16 229 (39.1)	5310 (47.4)	52 027 (55.5)	19 274 (42.0)	10 442 (42.5)	3460 (49.1)
Medicare	48 331 (31.0)	28 021 (35.2)	13 163 (31.7)	3629 (32.4)	31 950 (34.1)	16 130 (35.1)	7754 (31.6)	2279 (32.3)
Medicaid	10 964 (7.0)	12 464 (15.7)	7161 (17.2)	1492 (13.3)	7176 (7.7)	7411 (16.1)	4436 (18.1)	1056 (15.0)
Other	6791 (4.4)	7568 (9.5)	4979 (12.0)	764 (6.8)	2542 (2.7)	3107 (6.8)	1920 (7.8)	256 (3.6)
Received nephrology care for >12 mo before dialysis	57 310 (36.8)	24 033 (30.2)	10 669 (25.7)	3749 (33.5)	37 003 (39.5)	14 944 (32.5)	6945 (28.3)	2618 (37.1)

Abbreviations: BMI, body mass index (calculated as weight in kilograms divided by height in meters squared); ESKD, end-stage kidney disease.

^a^ Unless otherwise indicated, data are expressed as number (percentage) of patients. Percentages have been rounded and may not total 100.

### Temporal Trends in Racial/Ethnic Disparities in Receipt of at Least 12 Months of Predialysis Nephrology Care

During the study period, the unadjusted proportion of adults who received at least 12 months of predialysis nephrology care increased from the 2005 to 2007 to the 2014 to 2015 cohorts by 30.1% to 39.5% among White adults, 24.5% to 32.5% among Black adults, 21.2% to 28.3% among Hispanic adults, and 26.1% to 37.1% among Asian adults. However, the magnitude of racial/ethnic disparities did not improve during the study period (*P* > .10 for statistical interaction terms by race/ethnicity and year). In the 2005 to 2007 cohort, compared with receipt of at least 12 months of predialysis nephrology care among White adults, the aOR was 0.82 (95% CI, 0.80-0.84) among Black adults, 0.67 (95% CI, 0.65-0.69) among Hispanic adults, and 0.84 (95% CI, 0.80-0.89) among Asian adults; in the 2014 to 2015 cohort, the aOR was 0.76 (95% CI, 0.74-0.78) among Black adults, 0.61 (95% CI, 0.60-0.63) among Hispanic adults, and 0.90 (95% CI, 0.86-0.95) among Asian adults. Inferences from crude models were similar to inferences from adjusted models (Table 3). The full model is provided as eTable 1 in the Supplement. Inferences from additional multivariable logistic regression models examining racial/ethnic differences in receipt of predialysis care at any time before ESKD therapy were also similar to inferences from primary models (eTable 2 in the Supplement). The adjusted risk ratios estimated from modified Poisson regression models performed in sensitivity analyses are provided in eTable 3 in the Supplement.

**Table 3.** Temporal Trends in Racial/Ethnic Disparities in Receipt of at Least 12 Months of Predialysis Nephrology Care

Cohort year	Crude OR (95% CI)				Adjusted OR (95% CI)^a^			
	White	Black	Hispanic	Asian	White	Black	Hispanic	Asian
2005-2007	1 [Reference]	0.74 (0.72-0.75)	0.61 (0.59-0.63)	0.81 (0.77-0.85)	1 [Reference]	0.82 (0.80-0.84)	0.67 (0.65-0.69)	0.84 (0.80-0.89)
2008-2010	1 [Reference]	0.71 (0.69-0.72)	0.58 (0.57-0.60)	0.81 (0.78-0.85)	1 [Reference]	0.77 (0.76-0.79)	0.63 (0.61-0.65)	0.84 (0.81-0.88)
2011-2013	1 [Reference]	0.72 (0.71-0.73)	0.57 (0.56-0.59)	0.83 (0.80-0.86)	1 [Reference]	0.78 (0.76-0.79)	0.61 (0.59-0.62)	0.85 (0.81-0.88)
2014-2015	1 [Reference]	0.71 (0.70-0.73)	0.60 (0.58-0.61)	0.90 (0.86-0.94)	1 [Reference]	0.76 (0.74-0.78)	0.61 (0.60-0.63)	0.90 (0.86-0.95)

Abbreviation: OR, odds ratio.

^a^ Adjusted for differences in age (continuous), sex (male or female), body mass index (calculated as weight in kilograms divided by height in meters squared; ≤30.0 or >30.0), and end-stage kidney disease etiology (diabetes, hypertension, glomerular diseases, or other).

### Factors Associated With Racial/Ethnic Disparities in Receipt of Predialysis Nephrology Care

Exploratory mediation analysis findings from incremental multivariable logistic regression models (Table 4) suggest adjustments for differences in health insurance type were more strongly associated with slight attenuation of racial/ethnic disparities in receipt of at least 12 months of predialysis care among Black patients (aORs, 0.94 [95% CI, 0.93-0.94] in the 2005-2007 cohort vs 0.93 [95% CI, 0.93-0.94] in the 2008-2010 and 2011-2013 cohorts) and Hispanic patients (aORs, 0.89 [95% CI, 0.88-0.89] to 0.88 [95% CI, 0.88-0.89] for the 2005-2007 vs 2014-2015 cohorts) than adjustments for comorbid medical conditions, type of dialysis modality, or type of vascular access. However, even in fully adjusted models, our primary inferences remained the same: the magnitude of racial/ethnic disparities did not statistically significantly improve during the study period.

**Table 4.** Exploratory Mediation Analysis of Racial/Ethnic Disparities in Receipt of at Least 12 Months of Predialysis Nephrology Care^a^

Cohort	OR (95% CI)			
	White	Black	Hispanic	Asian
**Regression model 2**^b^				
2005-2007	1 [Reference]	0.90 (0.89-0.91)	0.84 (0.83-0.85)	0.99 (0.97-1.00)
2008-2010	1 [Reference]	0.90 (0.90-0.91)	0.81 (0.80-0.82)	0.98 (0.97-1.00)
2011-2013	1 [Reference]	0.91 (0.90-0.91)	0.83 (0.82-0.84)	0.98 (0.97-1.00)
2014-2015	1 [Reference]	0.92 (0.91-0.92)	0.86 (0.85-0.87)	0.99 (0.98-1.00)
**Regression model 3**^c^				
2005-2007	1 [Reference]	0.92 (0.92-0.93)	0.87 (0.86-0.88)	0.98 (0.96-0.99)
2008-2010	1 [Reference]	0.92 (0.92-0.93)	0.85 (0.84-0.85)	0.98 (0.96-0.99)
2011-2013	1 [Reference]	0.93 (0.92-0.93)	0.86 (0.86-0.87)	0.98 (0.97-0.99)
2014-2015	1 [Reference]	0.94 (0.93-0.95)	0.89 (0.89-0.90)	0.98 (0.97-1.00)
**Regression model 4**^d^				
2005-2007	1 [Reference]	0.94 (0.93-0.94)	0.89 (0.88-0.89)	1.02 (1.00-1.03)
2008-2010	1 [Reference]	0.93 (0.93-0.94)	0.85 (0.85-0.86)	1.01 (1.00-1.02)
2011-2013	1 [Reference]	0.93 (0.93-0.94)	0.87 (0.86-0.87)	1.01 (1.00-1.02)
2014-2015	1 [Reference]	0.94 (0.93-0.94)	0.88 (0.88-0.89)	1.01 (0.99-1.02)
**Regression model 5**^e^				
2005-2007	1 [Reference]	0.94 (0.94-0.95)	0.90 (0.89-0.91)	0.99 (0.98-1.00)
2008-2010	1 [Reference]	0.94 (0.93-0.95)	0.87 (0.86-0.88)	0.99 (0.98-1.00)
2011-2013	1 [Reference]	0.94 (0.94-0.95)	0.88 (0.88-0.89)	0.99 (0.98-1.00)
2014-2015	1 [Reference]	0.95 (0.94-0.95)	0.90 (0.90-0.91)	0.98 (0.97-1.00)

Abbreviation: OR, odds ratio.

^a^ All multivariable models were adjusted for differences in age (continuous), sex (male or female), body mass index (calculated as weight in kilograms divided by height in meters squared; ≤30.0 or >30.0), and end-stage kidney disease etiology (diabetes, hypertension, glomerular diseases, or other).

^b^ Adjusted for comorbid conditions (ie, cancer, atherosclerotic heart disease, congestive heart failure, diabetes, hypertension, chronic obstructive pulmonary disease, or peripheral vascular disease).

^c^ Adjusted for type of dialysis modality and type of vascular access.

^d^ Adjusted for health insurance type.

^e^ Fully adjusted for all potential confounders and mediators.

## Discussion

In this national registry study of more than 1 million adults who initiated maintenance dialysis treatment in the United States from 2005 to 2015, 31.1% of patients received at least 12 months of predialysis nephrology care. In addition, racial/ethnic disparities in receipt of at least 12 months of predialysis nephrology care did not substantially improve during the study period. Secondary study findings from exploratory mediation analyses suggest that racial/ethnic differences in health insurance type may be more strongly associated with slight attenuation of racial/ethnic disparities in predialysis nephrology care than differences in comorbid medical conditions, dialysis type, or vascular access type among Black and Hispanic patients.

Findings from our study suggest that national strategies designed to target racial/ethnic disparities are needed to achieve equity in access to predialysis nephrology care. Potential strategies may include national efforts to enhance collaborations between primary care providers and nephrologists, particularly for members of racial/ethnic minority groups.^4,14,15^ For instance, the United Kingdom has successfully implemented a primary care–based CKD management program that has resulted in improved CKD care and education, dissemination of new and existing educational tools, and early education of primary care trainees.^15^ Similar integrated care models could be applied to clinical settings in the United States; in particular, national strategies are needed to improve primary care clinicians’ capacities to deliver optimal CKD care and comanagement of patients with nephrologists (eg, team-based decision support). Integrated care coordination will be vital to achieving the HP2020^12^ goals of eliminating health care disparities and substantially increasing the proportion of patients with CKD receiving at least 12 months of predialysis care from a nephrologist.

We also found that Black and Hispanic patients are less likely to receive at least 12 months of predialysis nephrology care than White patients, independent of differences in clinical and demographic factors. Although prior studies reported that women are less likely to receive pre-ESKD nephrology care owing to their lower prevalence of CKD, slower progression, and better clinical outcomes of CKD,^9^ we found that racial/ethnic disparities are associated with receipt of predialysis nephrology care by both male and female patients. We also found that racial/ethnic differences in health insurance type did not fully explain disparities in predialysis nephrology care. These novel findings build on and expand prior work examining the association of health insurance status with likelihood of receiving predialysis specialty care.^7,8,9,10,11^ In a prior study,^10^ authors concluded that patients in the US Department of Veterans Affairs system are more likely to receive pre-ESKD nephrology care than the general population outside the Veterans Affairs health care system because of their greater access to subspecialty care, use of electronic health records, case management, and integrated clinical guidelines for early recognition and management.

### Strengths and Limitations

Our study has several strengths. The first is the ability to comprehensively analyze a decade of national data from the well-characterized population of adults who initiated treatment for ESKD in the United States. A second strength of the study is the ability to account for differences in many important characteristics that might confound or potentially mediate observed associations.

This study also had limitations, one of which is that the primary study outcome is subject to the accuracy of physician-provided retrospective data documented on the ESRD Medical Evidence Report Form CMS 2728, as well as the inability to further subcategorize the broad racial/ethnic categories available in the national USRDS registry (ie, Hispanic/Latino, Asian, Black/African American, and White). Another limitation is that the study was unable to account for individual- or household-level patient income. The future availability of individual patient or household income in national registries would allow us to better delineate the extent to which trends in socioeconomic measures may contribute to changes in predialysis care disparities over time.

## Conclusions

In this national study of more than 1 million US adults with ESKD, racial and ethnic disparities in receipt of at least 12 months of predialysis nephrology care did not substantially improve from 2005 to 2015. These findings suggest that national strategies to address disparities in predialysis health care are needed.

## Notes

Supplement. **eTable 1.** Full Model: Racial/Ethnic Disparities in Receipt of at Least 12 Months of Predialysis Nephrology Care **eTable 2.** Sensitivity Analysis: Racial/Ethnic Disparities in Receipt of Predialysis Nephrology Care at Any Time Before ESKD Therapy From 2005-2007 to 2014-2015 **eTable 3.** Sensitivity Analysis: Racial/Ethnic Disparities in Receipt of at Least 12 Months of Predialysis Nephrology Care
