# Supplementary material for: National Trends in the Association of Race and Ethnicity With Predialysis Nephrology Care in the United States From 2005 to 2015
Source: JAMA Netw Open. 2020 Aug 27;3(8):e2015003. doi: 10.1001/jamanetworkopen.2020.15003 (PMC7453308; doi:10.1001/jamanetworkopen.2020.15003)
Supplement: Supplement. — eTable 1. Full Model: Racial/Ethnic Disparities in Receipt of at Least 12 Months of Predialysis Nephrology Care eTable 2. Sensitivity Analysis: Racial/Ethnic Disparities in Receipt of Predialysis Nephrology Care at Any Time Before ESKD Therapy From 2005-2007 to 2014-2015 eTable 3. Sensitivity Analysis: Racial/Ethnic Disparities in Receipt of at Least 12 Months of Predialysis Nephrology Care [file jamanetwopen-3-e2015003-s001.pdf]

Supplementary Online Content

Purnell TS, Bae S, Luo X, et al. National trends in the association of race and ethnicity with predialysis nephrology care in the United States from 2005 to 2015. *JAMA Netw Open*. 2020;3(8):e2015003. doi:10.1001/jamanetworkopen.2020.15003

eTable 1. Full Model: Racial/Ethnic Disparities in Receipt of at Least 12 Months of Predialysis Nephrology Care

eTable 2. Sensitivity Analysis: Racial/Ethnic Disparities in Receipt of Predialysis Nephrology Care At Any Time Before ESKD Therapy From 2005-2007 to 2014-2015

eTable 3. Sensitivity Analysis: Racial/Ethnic Disparities in Receipt of at Least 12 Months of Predialysis Nephrology Care

This supplementary material has been provided by the authors to give readers additional information about their work.

©2020 Purnell TS et al. *JAMA Network Open*.

eTable 1. Full Model: Racial/Ethnic Disparities in Receipt of at Least 12 Months of Predialysis Nephrology Care

	**Coefficient**	**95% CI**
Race		
White	Reference	
Black	-0.299	(-0.319, -0.280)
Hispanic	-0.524	(-0.549, -0.499)
Asian	-0.034	(-0.084, 0.015)
Year of transplant, per year	0.040	(0.038, 0.042)
Race-Year interaction		
Black	-0.001	(-0.004, 0.003)
Hispanic	-0.003	(-0.008, 0.001)
Asian	0.005	(-0.004, 0.013)
Age	0.004	(0.004, 0.004)
Female	0.045	(0.036, 0.053)
BMI category		
< 18.5 kg/m2	-0.191	(-0.214, -0.168)
18.5 - 35 kg/m2	Reference	
>35 kg/m2	0.054	(0.043, 0.065)
Cause of ESRD		
Diabetes	Reference	
Glomerulonephritis	0.131	(0.112, 0.150)
Hypertension	-0.330	(-0.341, -0.320)
Others	-0.665	(-0.676, -0.654)
Constant	0.687	(0.664, 0.711)

Shows the full model results from the multivariable logistic regression model examining temporal trends in racial/ethnic disparities in receipt of at least 12 months of pre-dialysis nephrology care.

©2020 Purnell TS et al. *JAMA Network Open*.

eTable 2. Sensitivity Analysis: Racial/Ethnic Disparities in Receipt of Predialysis Nephrology Care At Any Time Before ESKD Therapy From 2005-2007 to 2014-2015

Cohort Year	Crude Odds Ratios (95% CI)			
	White	Black	Hispanic	Asian
2005-2007	Reference	0.75 (0.74-0.77)	0.66 (0.65-0.68)	1.03 (0.98-1.08)
2008-2010	Reference	0.76 (0.74-0.77)	0.61 (0.60-0.63)	1.03 (0.99-1.08)
2011-2013	Reference	0.74 (0.73-0.76)	0.62 (0.60-0.63)	1.05 (1.00-1.09)
2014-2015	Reference	0.74 (0.73-0.76)	0.65 (0.63-0.67)	1.09 (1.02-1.15)
	**Adjusted Odds Ratios (95% CI)**			
**Cohort Year**	**White**	**Black**	**Hispanic**	**Asian**
2005-2007	Reference	0.82 (0.81-0.84)	0.70 (0.68-0.72)	1.05 (1.00-1.11)
2008-2010	Reference	0.81 (0.80-0.83)	0.63 (0.62-0.65)	1.03 (0.99-1.08)
2011-2013	Reference	0.78 (0.77-0.80)	0.62 (0.61-0.64)	1.03 (0.99-1.08)
2014-2015	Reference	0.77 (0.75-0.79)	0.64 (0.62-0.66)	1.05 (0.99-1.11)

Shows the crude odds ratios (ORs) from unadjusted logistic regression models and the adjusted ORs from multivariable logistic regression models* comparing racial/ethnic disparities in receipt of pre-dialysis nephrology care *at any time* prior to initiation of ESKD therapy by time cohort (2005-2007, 2008-2010, 2011-2013, and 2014-2015).

*Multivariable models were adjusted for differences in age (continuous), sex (male, female), body mass index (≤30 kg/m^2, >30 kg/m^2), ESKD etiology (diabetes, hypertension, glomerular diseases, other).

©2020 Purnell TS et al. *JAMA Network Open*.

eTable 3. Sensitivity Analysis: Racial/Ethnic Disparities in Receipt of at Least 12 Months of Predialysis Nephrology Care

Cohort Year	Crude Risk Ratios (95% CI)			
	White	Black	Hispanic	Asian
2005-2007	Reference	0.91 (0.90-0.91)	0.86 (0.86-0.87)	1.01 (0.99-1.03)
2008-2010	Reference	0.91 (0.90-0.92)	0.84 (0.83-0.84)	1.01 (1.00-1.02)
2011-2013	Reference	0.91 (0.91-0.92)	0.86 (0.85-0.86)	1.01 (1.00-1.02)
2014-2015	Reference	0.92 (0.92-0.93)	0.88 (0.88-0.89)	1.02 (1.01-1.03)
	**Adjusted Risk Ratios (95% CI)**			
**Cohort Year**	**White**	**Black**	**Hispanic**	**Asian**
2005-2007	Reference	0.91 (0.90-0.91)	0.85 (0.84-0.85)	1.00 (0.98-1.01)
2008-2010	Reference	0.91 (0.90-0.91)	0.82 (0.81-0.83)	0.99 (0.98-1.01)
2011-2013	Reference	0.91 (0.91-0.92)	0.84 (0.83-0.84)	0.99 (0.98-1.01)
2014-2015	Reference	0.92 (0.92-0.93)	0.87 (0.86-0.87)	1.00 (0.99-1.02)

Shows the crude and adjusted risk ratios from ***modified Poisson regression models*** comparing racial/ethnic disparities in receipt of at least 12 months of pre-dialysis nephrology care by time cohort (2005-2007, 2008-2010, 2011-2013, and 2014-2015).

*Adjusted models were adjusted for differences in age (continuous), sex (male, female), body mass index (≤30 kg/m^2, >30 kg/m^2), ESKD etiology (diabetes, hypertension, glomerular diseases, other).

©2020 Purnell TS et al. *JAMA Network Open*.
